# Hedgehog/GLI signaling in hematopoietic development and acute myeloid leukemia—From bench to bedside

**DOI:** 10.3389/fcell.2022.944760

**Published:** 2022-08-05

**Authors:** Suzana Tesanovic, Peter W. Krenn, Fritz Aberger

**Affiliations:** Department of Biosciences and Medical Biology, Cancer Cluster Salzburg, Paris-Lodron University of Salzburg, Salzburg, Austria

**Keywords:** acute myeloid leukemia, hedgehog (Hh) signaling, GLI proteins, cancer stem cell, leukemic stem cell, tumor microenvironment, combination therapy, smoothened inhibitor

## Abstract

While the underlying genetic alterations and biology of acute myeloid leukemia (AML), an aggressive hematologic malignancy characterized by clonal expansion of undifferentiated myeloid cells, have been gradually unraveled in the last decades, translation into clinical treatment approaches has only just begun. High relapse rates remain a major challenge in AML therapy and are to a large extent attributed to the persistence of treatment-resistant leukemic stem cells (LSCs). The Hedgehog (HH) signaling pathway is crucial for the development and progression of multiple cancer stem cell driven tumors, including AML, and has therefore gained interest as a therapeutic target. In this review, we give an overview of the major components of the HH signaling pathway, dissect HH functions in normal and malignant hematopoiesis, and specifically elaborate on the role of HH signaling in AML pathogenesis and resistance. Furthermore, we summarize preclinical and clinical HH inhibitor studies, leading to the approval of the HH pathway inhibitor glasdegib, in combination with low-dose cytarabine, for AML treatment.

## Introduction

Hematopoietic stem cells (HSCs) are the apex of the hierarchically organized blood cell production system giving rise to multipotent hematopoietic progenitor cells (HPCs), unipotent HPCs, and finally blood effector cells. Within the supporting bone marrow microenvironment HSCs are maintained in a delicate balance between self-renewal and differentiation to ensure life-long steady-state hematopoiesis and HPC and effector cell replenishment upon, e.g., blood loss or infections (Krenn et al., 2022). Upon transplantation into a lethally irradiated or immunosuppressed recipient mouse a single HSC, characterized by lineage negativity and high sca-1, high c-kit, low/absent CD34 and absent CD38 expression, can fully reestablish the hematopoietic system (Osawa et al., 1996). Similarly, transplantation of acute myeloid leukemia (AML) patient-derived CD34^+^CD38^−^ cells results in the establishment of an AML-like disease including the development of leukemic blasts (Bonnet and Dick, 1997). These seminal studies suggested a residual and skewed hematopoietic hierarchy in AML originating from a transformed leukemic stem cell (LSC). Importantly, tumor initiating cancer stem cells (CSCs), which possess the capacity to self-renew, generate all tumor cell populations, and cause relapse after chemotherapy, were also shown to exist in many other tumor entities, such as breast, skin, brain and gastrointestinal cancers (Batlle and Clevers, 2017). Due to their partially overlapping functionality, it was suggested that normal and cancer stem cells share common signaling pathways required for their maintenance and pro-longed survival (Koury et al., 2017). In this review we will specifically introduce and discuss the Hedgehog (HH) signaling pathway as such a shared but differentially utilized pathway in HSCs and AML-LSCs and elaborate on current treatment approaches aiming to abrogate HH signaling in leukemic cells.

## The Hedgehog signaling pathway

The HH signaling pathway, initially discovered as a regulator of segment polarity in the fruit fly, is a highly conserved signaling cascade that regulates several aspects of embryonic development while being largely silenced in adult tissues except for i.e., stem and progenitor cell maintenance, metabolic control, inflammatory processes and tissue repair after injury (Nüsslein-Volhard and Wieschaus, 1980; Echelard et al., 1993; Ingham and McMahon, 2001; Beachy et al., 2004; Pospisilik et al., 2010; Teperino et al., 2012; Furmanski et al., 2013; Lau et al., 2021). Aberrant pathway activation has been associated with several human cancers, ranging from medulloblastoma to basal cell carcinoma (BCC) and hematological malignancies (reviewed in Teglund and Toftgård, 2010 and Abraham and Matsui, 2021). Oncogenic HH signaling contributes to several hallmarks of cancer and supports initiation, progression and metastasis of various tumor entities by impacting on the cancer cells themselves as well as by modulating the tumor supporting microenvironment (Gailani et al., 1996; Das et al., 2009; Alexaki et al., 2010; Hanahan and Weinberg, 2011; Hanna and Shevde, 2016; Riobo-Del Galdo et al., 2019; Zhang et al., 2021).

### Canonical Hedgehog signaling

**FIGURE 1:**
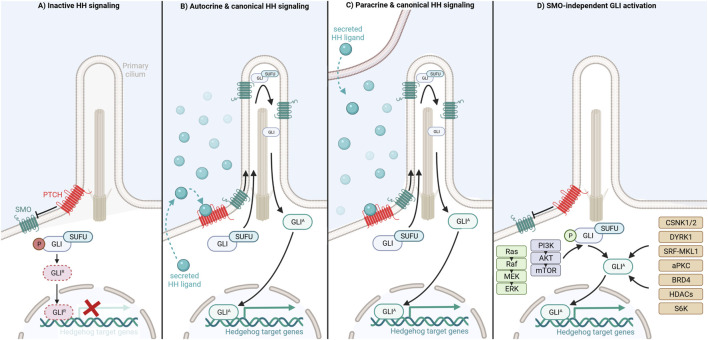
Activation and distinct regulatory mechanisms of Hedgehog/GLI signaling modes.

In mammals canonical HH signaling is initiated through binding of one of three secreted HH ligands, Sonic hedgehog (SHH), Indian hedgehog (IHH) or Desert hedgehog (DHH), to the twelve-transmembrane domain receptor Patched (PTCH) (Ingham and McMahon, 2001). In an unliganded state, PTCH is located at the base of the primary cilium, a slim hair-like organelle indispensable for canonical vertebrate HH signaling present on the surface of almost all mammalian cells, including normal blood cells (Rosenbaum and Witman, 2002; Goetz and Anderson, 2010; Singh et al., 2016). PTCH exerts its inhibitory function by antagonizing the signal transducer Smoothened (SMO), a seven transmembrane protein and member of the G-protein coupled receptor superfamily, allowing the phosphorylation of the GLI transcription factors, a process facilitated by the GLI-binding protein Suppressor of Fused (SUFU) (Ingham and McMahon, 2001; Humke et al., 2010). Subsequently, phosphorylated GLI is targeted for selective proteasomal degradation resulting in a truncated repressor form of GLI (GLI^R^), which translocates to the nucleus and inhibits HH-induced target gene expression (Humke et al., 2010; Gulino et al., 2012) (Figure 1A).

Upon binding of a HH ligand to PTCH, PTCH is inactivated by internalization in an endosome dependent manner and degradation in lysosomes (Incardona et al., 2000; Ingham and McMahon, 2001). Removal of PTCH from the primary cilium cancels PTCH-mediated repression of SMO allowing SMO activation and translocation into the primary cilium (Teglund and Toftgård, 2010; Chen et al., 2011). Activated SMO promotes accumulation of GLI at the tip of the primary cilium, GLI dissociation from SUFU and generation of full-length GLI activator (GLI^A^) forms. Eventually, GLI^A^ shuttles to the nucleus where it acts as a transcription factor and induces the expression of HH target genes including regulators of cell differentiation, survival and proliferation as well as components of the HH/GLI pathway itself, such as GLI1 and PTCH1 (Humke et al., 2010; Tukachinsky et al., 2010; Aberger and Ruiz i Altaba, 2014). Importantly, HH ligands can be produced by, act on and activate HH signaling in the same cell (autocrine HH signaling) or neighboring cells (paracrine HH signaling) (Fan et al., 2004; Sanchez et al., 2004; Bhowmick and Moses, 2005; Dierks et al., 2007; Yuan et al., 2007; Hegde et al., 2008; Yauch et al., 2008; Tian et al., 2009; Amakye et al., 2013; Liu et al., 2014) (Figures 1B,C). The transcriptional outcome of HH signaling depends on the ratio of GLI^A^ and proteolytically processed GLI^R^ within the nucleus. Activator and repressor functions are fulfilled to varying degrees by the three different mammalian GLIs, namely GLI1, GLI2, and GLI3. While GLI1 solely acts as a transcriptional activator, GLI2 functions as both strong transcriptional activator and modest repressor if proteolytically processed. By contrast, GLI3 is mainly considered as transcriptional repressor generated by proteolytic processing, though GLI3 full-length protein can also induce target gene expression in a context-dependent manner (Sasaki et al., 1999; Park et al., 2000; Bai and Joyner, 2001; Buttitta et al., 2003; Pan et al., 2006; Ruiz i Altaba et al., 2007; Hui and Angers, 2011; Li et al., 2011).

### SMO-independent Hedgehog signaling

GLI^A^-dependent transcription can additionally be induced and upregulated in cancer cells in a SMO-independent manner via crosstalk with and integration of oncogenic signaling and epigenetic cues (Teperino et al., 2014; Doheny et al., 2020). As shown for melanoma and colon cancer cells, the constitutive activation of RAS-RAF-MEK-ERK signaling results in increased nuclear localization and/or transcriptional activity of GLI1 and/or GLI2 (Stecca et al., 2007; Mazumdar et al., 2011). Similarly, expression, nuclear localization, protein stability and transcriptional activity of the GLI transcription factors are enhanced by the PI3K/AKT/mTOR pathway in, for instance, renal cell carcinoma, adeno carcinoma, lung squamous cell carcinoma, pancreatic cancer, and ovarian cancer cells (Kebenko et al., 2015; Zhou et al., 2016; Aberger et al., 2017; Kasiri et al., 2017; Singh et al., 2017). As shown in Figure 1D, several druggable non-canonical HH effectors have been identified as positive regulators of GLI proteins such as casein kinases (CSNK1, CSNK2), DYRK1, atypical PKC, SRF-MKL1, BRD4, S6K and class-I histone deacetylases. Pharmacological targeting of these effectors provides promising opportunities for the treatment of HH/GLI-associated cancers in combination with established HH-antagonists or in settings with *a priori* or acquired SMO-inhibitor resistance (Mao et al., 2002; Canettieri et al., 2010; Wang et al., 2012; Atwood et al., 2013; Coni et al., 2013; Tang et al., 2014; Gruber et al., 2016; Singh et al., 2017; Gruber et al., 2018; Purzner et al., 2018; Whitson et al., 2018; Peer et al., 2021) (for more details see in-depth reviews of Aberger and Ruiz i Altaba, 2014; Singh and Lauth, 2017; Peer et al., 2019; Pietrobono et al., 2019).

## Hedgehog signaling in normal hematopoiesis

Although the first link between HH signaling and hematopoiesis was reported 2 decades ago, a well-defined role for HH signaling within the hematopoietic system has so far not been established (Bhardwaj et al., 2001; Lim and Matsui, 2010; Mar et al., 2011; Aberger et al., 2012). The vertebrate hematopoietic system develops in three spatially and temporally distinguishable waves during embryonic development: 1) primitive hematopoiesis, which takes place in mesoderm-derived yolk-sac blood islands and produces embryonic erythrocytes; 2) pro-definitive hematopoiesis, which originates from the yolk sac hemogenic endothelium producing bipotent HPCs required for blood cell production until birth; 3) definitive hematopoiesis, which originates from hemogenic endothelium of the yolk sac, placenta and/or aorta-gonad-mesonephros region giving rise to HSCs required for multilineage hematopoiesis. Newly generated HSCs initially seed into placenta and fetal liver for expansion and maturation and subsequently migrate to bone marrow for their maintenance ensuring life-long self-renewal and balanced blood cell production (reviewed in Krenn et al., 2022).

Already during early gastrulation HH-signaling paves the way for the initiation of hematopoiesis. Using mouse embryonic explants, it was shown that visceral-endoderm secreted IHH upregulates the expression and activity of PTCH1, SMO and GLI1 within the epiblast, thereby contributing to the generation of hemogenic and vasculogenic mesodermal cells (Farrington et al., 1997; Dyer et al., 2001). Upon antibody-based inhibition or genetic deletion of either IHH or SMO these mesodermal cells were unable to form yolk sac blood islands required for early embryonic erythropoiesis and vascularization (Byrd et al., 2002). These severe defects are only partially recapitulated *in vivo* as only 50% of IHH-deficient embryos die at mid-gestation (St-Jacques et al., 1999; Dyer et al., 2001). This suggests that loss of IHH is compensated by alternative HH ligands, non-canonical pathway activation or redundancy with other developmental pathways *in vivo*.

Possibly due to masking hematopoiesis-independent defects and/or early embryonic lethality observed in many conventional HH knockout models (Chiang et al., 1996; Mo et al., 1997; St-Jacques et al., 1999; Zhang et al., 2001; Cooper et al., 2005), we do not clearly understand the role for HH signaling in the generation and function of erythro-myeloid progenitors or HSCs prior to birth in mouse or human *in vivo*. However, considering that HH-mutant zebrafish embryos do not establish HSCs (Gering and Patient, 2005) and *ex vivo* treatment of AGM tissue explants with SHH or IHH increases HSC activity (Peeters et al., 2009), *in vivo* studies taking advantage of now available conditional knockout mouse models allowing for cell-type specific ablation of HH family members during development are envisioned and required for clarification.

Hematopoietic stem and progenitor cells (HSPCs) isolated from human newborn cord blood, frequently termed primitive HSPCs, express PTCH1, SMO, SHH, IHH, GLI1, GLI2 and GLI3 mRNA (Bhardwaj et al., 2001; Kobune et al., 2004). While function-blocking antibodies targeting HH ligands reduced *in vitro* proliferation and differentiation of primitive HSPCs, treatment with recombinant SHH or DHH or co-culture with bone marrow-derived stromal cells secreting IHH increased their *in vitro* proliferation and *in vivo* repopulation capacity upon transplantation into nonobese-severe-combined immunodeficient (NOD-SCID) mice.

In line with these observations, adult mice harboring a heterozygous *Ptch1* deletion (*Ptch*1^+/−^), which results in increased HH pathway activity, revealed an expansion of HPCs in the bone marrow under steady-state conditions compared to wild-type littermates. When treated with 5-fluorouracil, which depletes HPCs and effector cells, thereby leading to a strong activation and proliferation of HSCs (Lerner and Harrison, 1990; Wilson et al., 2008), the peripheral blood cell pool of *Ptch1* ^+/−^ mice recovered faster, suggesting increased HSC regenerative potential. However, as shown by serial transplantation of *Ptch1* ^+/−^ HSPCs, the increased HSPC proliferation resulted in reduced HSC self-renewal and maintenance (Trowbridge et al., 2006; Dierks et al., 2008). Inducible deletion of the *Ptch1* gene in adult mice also increased HSPC proliferation rates but additionally resulted in a rapid decrease in T and B cell numbers, suggesting possible differentiation defects (Uhmann et al., 2007). In agreement with adult HSPCs not expressing the HH downstream transcription factors GLI1, GLI2 or GLI3 (Gao et al., 2009), PTCH1-mediated HSPC maintaining signals are not HSPC-intrinsic but provided by the HSPC supporting bone marrow microenvironment (Uhmann et al., 2007; Siggins et al., 2009).

Due to the embryonic lethality of Smo^−/−^ mice, the role of SMO for adult hematopoiesis was analyzed in conditional knockout mouse models or by transplantation of fetal liver-derived Smo^−/−^ HSPCs into irradiated recipient mice. These studies have, however, produced conflicting results. While conditional deletion of *Smo* in hematopoietic and endothelial cells using the *Vav-Cre* transgene significantly reduced the numbers of long-term repopulating HSCs in the bone marrow (Zhao et al., 2009), conditional deletion of *Smo* in hematopoietic cells, osteoblasts, and perivascular MSCs but not bone marrow endothelial cells using the inducible *Mx1-Cre* transgene did not alter adult hematopoiesis and HSC function under steady-state or stress (Gao et al., 2009; Hofmann et al., 2009). Similarly, hematopoiesis was restored normally after transplantation of fetal liver-derived Smo^−/−^ HSPCs into sublethally irradiated recipient mice (Dierks et al., 2008). Reconciling this discrepancy, a unifying hypothesis could be that SMO-mediated HH signaling within endothelial cells is crucial for HSPC maintenance in bone marrow. Of note, the pharmacological inhibition of SMO with a high-affinity antagonist did not impair *in vitro* nor *in vivo* HSPC differentiation and proliferation under steady-state conditions (Hofmann et al., 2009). However, SMO-antagonist treated HSCs were not tested for their repopulation capacity in transplantation experiments.

Gli1^null^ mice, although viable and presenting normal peripheral blood counts, display reduced HSPC proliferation, impaired myeloid differentiation, and defective 5-FU- or G-CSF-induced stress hematopoiesis. The increased quiescence of the HSPC compartment resulted in increased HSC-mediated long-term repopulation of lethally irradiated recipients upon transplantation (Merchant et al., 2010). However, considering that neither GLI1 mRNA nor protein have been detected in adult HSPCs (Gao et al., 2009), the data suggests that direct HH signaling is restricted to cells of the bone marrow microenvironment, which support HSPCs.

Together, these studies highlight a so far unappreciated role of microenvironment-mediated HH signaling for HSPC development and function. Future work will not only have to dissect the individual contribution of distinct bone marrow microenvironment components and HSPCs to HH signaling but also clarify if and how HH signaling contributes to adult hematopoiesis compared to embryonic hematopoiesis.

## Acute myeloid leukemia

Acute myeloid leukemia (AML) is a heterogeneous clonal hematologic disorder characterized by multiple cytogenetic and molecular abnormalities leading to the production and accumulation of undifferentiated myeloid progenitors, so-called leukemic blasts, which displace the normal hematopoietic system in the bone marrow (Zhou et al., 2016). The establishment of LSCs, the source of leukemic blasts, and coinciding development of AML is a multistep process based on acquired genetic and epigenetic alterations within normal HSCs or early HPCs. Early mutations, often affecting epigenetic regulators, enhance the self-renewal potential of HSCs while simultaneously impairing differentiation causing the clonal expansion of pre-leukemic HSCs. At later disease stages mutations affect and deregulate crucial signaling pathways involved in proliferation and differentiation, thereby contributing to the full transformation of HSCs into LSCs (Corces-Zimmerman et al., 2014; Shlush et al., 2014; Vetrie et al., 2020). Within the bone marrow, LSCs hijack and remodel HSC niches to ensure not only LSC maintenance and support (Behrmann et al., 2018) but also chemoresistance (Shlush et al., 2014; Thomas and Majeti, 2017; Boyd et al., 2018). While initial studies suggested that AML-LSCs are restricted to the rare quiescent CD34^+^CD38^−^ cell population, follow-up studies demonstrated that, especially in relapsed AML, LSCs are present within all bone marrow HSPC populations and, in part, highly proliferative (Iwasaki et al., 2015; Ho et al., 2016; Pollyea and Jordan, 2017). This phenotypic heterogeneity most likely reflects individual LSC subpopulations contributing to chemoresistance and relapse.

The early clinical manifestation of AML is directly attributable to the loss of functional hematopoietic effector cells with patients exhibiting fatigue, pallor, anemia, susceptibility to infections, easy bruising, or hemorrhage. Secondary organ infiltration of leukemic cells provokes the development of additional symptoms, such as splenomegaly, hepatomegaly, or lymphadenopathy (Löwenberg et al., 1999; Redaelli et al., 2003). AML is mainly a disease of the elderly with the median age at diagnosis being 68 years (Shallis et al., 2019). Besides increasing age, risk factors for the development of AML include inherited genetic disorders, elevated white blood cell count, preceding cytotoxic therapy, preceding hematologic disorders and genetic alterations (Dohner et al., 2015). Once diagnosed with AML, the 5-years survival rate is ∼29% across all AML patients and drops to ∼7% in AML patients aged 65 years and above (SEER-database, 2021). The individual patient survival, however, strongly depends on the tumor driving genetic alterations, which where therefore incorporated into the currently valid World Health Organization (WHO) and European LeukemiaNet (ELN) patient classifications and treatment recommendations for AML (Arber et al., 2016; Döhner et al., 2017; Hwang, 2020). Whereas cytogenetic profiling of large structural chromosomal abnormalities remains the backbone of AML patient classification, detection of recurrent mutations in AML, facilitated by easier access to next-generation sequencing approaches, has added an additional layer of heterogeneity among patients. The most frequently reported genetic abnormalities in AML affect the fms like tyrosine kinase 3 (FLT3), nucleophosmin (NPM1), DNA methyltransferase 3A (DNMT3A), isocitrate dehydrogenase 1 or 2 (IDH1, IDH2), Neuroblastoma RAS viral oncogene homolog/Kirsten rat sarcoma viral oncogene homolog (NRAS/KRAS), runt-related transcription factor 1 (RUNX1), Tet methylcytosine dioxygenase 2 (TET2), p53, CCAAT/enhanced binding protein α (CEBPA), and/or mixed-lineage leukemia (MLL) genes (Falini et al., 2005; Marcucci et al., 2011; Ferrara and Schiffer, 2013; Ley et al., 2013). Mutations affecting FLT3, NPM1 and IDH1/2 are associated with increased leukemic cell proliferation and survival by upregulation of JAK/STAT, PI3K/AKT and/or MEK/ERK signaling (Brandts et al., 2005; Breitenbuecher et al., 2009; Marcucci et al., 2011; Heydt et al., 2018), activation of several HOX genes ensuring a stem cell-like phenotype (Alcalay et al., 2005; Verhaak et al., 2005; Heath et al., 2017) and altered genome-wide or gene-specific DNA methylation, respectively (Ley et al., 2010; Im et al., 2014; Qu et al., 2014). Importantly, mutations may co-occur or exclude each other, thereby individually or in interplay modulating AML pathways contributing to disease progression or resistance (Ley et al., 2013).

The standard induction therapy for young and/or fit AML patients has remained largely unchanged for the last decades and consists of an intense chemotherapy with 7 days of cytarabine and 3 days of an anthracycline, such as daunorubicin or idarubicin (7 + 3 regimen), resulting in about 70% of patients <60 years and less than 50% of patients >60 years achieving complete remission (CR) (Wiernik et al., 1992; Ferrara and Schiffer, 2013; Aberger et al., 2017). Once CR is achieved, consolidation therapy is mandatory to eradicate residual LSCs and avoid relapse (Cassileth et al., 1988; Ferrara and Schiffer, 2013). However, considering the heterogeneous nature of AML, a single treatment approach cannot target all AML subtypes. Therefore, more selective and personalized therapies incorporating drugs targeting mechanisms induced by recurrent AML mutations have been studied in various clinical trials and led to the approval of AML treatment regimens including hypomethylating agents (HMAs; azacitidine, decitabine), pro-apoptotic agents (venetoclax), FLT3 kinase inhibitors (gilteritinib, midostaurin, sorafenib, quizartinib, crenolanib), and IDH inhibitors (ivosidinib, enasidinib). Recently, the first HH inhibitor glasdegib was approved for combination treatment with low-dose cytarabine for AML patients who are not eligible for high-dose chemotherapy. The impressive therapeutic effect of this combination therapy underscores the clinically relevant role of HH/GLI signaling in this lethal form of leukemia (Kantarjian et al., 2021).

## Hedgehog signaling in AML

Groundwork establishing HH signaling as a therapeutic target in AML include multiple studies investigating the expression of HH components in primary patient-derived whole mononuclear leukemic cells, CD34^+^ cells and/or bone marrow tissue. Consistent with AML heterogeneity, it was shown that the core components that mediate the HH signal response, PTCH1, SMO, GLI1, GLI2, and GLI3, are differentially expressed among AML patient samples (Bai et al., 2008; Kobune et al., 2009; Kobune et al., 2012; Wellbrock et al., 2015; Chaudhry et al., 2017). Noteworthy, increased expression of GLI2 was associated with the presence of FLT3 mutations and correlated with a significantly shortened event-free, relapse-free, and overall AML patient survival (Wellbrock et al., 2015). This finding suggests that in the context of continuous FLT3 activity GLI2 acts as a tumor promoting transcriptional activator. Consistently, AML patients harboring a high LSC frequency, identified by a high amount of GPR56 positive cells (Pabst et al., 2016), presented increased HH signaling and GLI2 activity (He et al., 2022). Similarly, increased GLI1 expression has been shown to correlate with reduced overall AML patient survival (Zhou et al., 2021).

**FIGURE 2:**
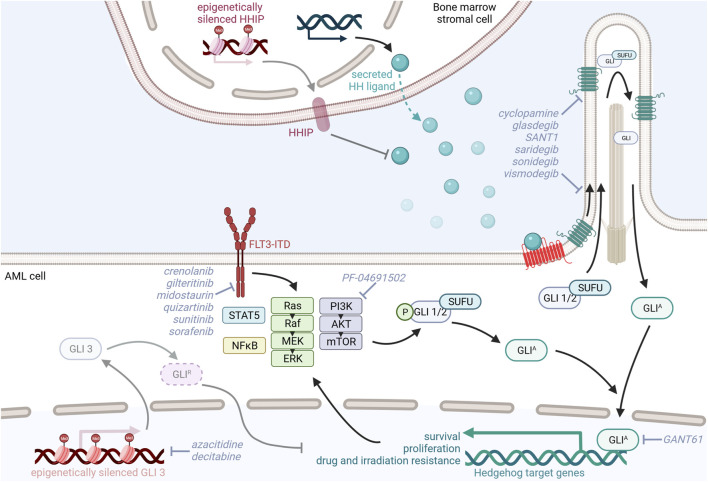
Model of oncogenic Hedgehog/GLI signaling in AML and its possible therapeutic targeting.

Studies investigating the expression of HH ligands have produced conflicting results. Whereas multiple studies were able to detect SHH and/or IHH mRNA in patient-derived leukemic cells (Bai et al., 2008; Kobune et al., 2009; Kobune et al., 2012; Chaudhry et al., 2017), a study by Wellbrock and others could neither detect SHH nor IHH or DHH transcripts within the leukemic cell fraction. However, Wellbrock and others convincingly showed that DHH is produced and shed into the blood by bone marrow endothelial cells and osteoblasts (Wellbrock et al., 2015). Likewise, Kobune et al. show that AML bone marrow stroma cells upregulate expression of IHH and downregulate expression of HH-interacting protein (HHIP), a negative HH regulator and transcriptional target of HH signaling, to support leukemic cell proliferation. Pretreatment with azacitidine induced demethylation of the HHIP gene, partially restored HHIP expression, and reduced the leukemia promoting effect of the primary AML stromal cells (Chuang and McMahon, 1999; Kobune et al., 2012) (Figure 2). Taken together, these datasets suggest a paracrine and tumor promoting interaction between leukemic cells and the bone marrow microenvironment via the HH signaling pathway.

### Preclinical *in vitro* studies targeting Hedgehog signaling in AML

Functionally, several *in vitro* studies have shown a correlation between HH signaling and AML cell resistance to chemotherapy and radiation. Inhibition of HH signaling using cyclopamine, GANT61, recombinant HHIP or anti-hedgehog neutralizing antibodies resulted in increased apoptosis, reduced proliferation, and restored chemosensitivity to cytarabine of CD34^+^ but not CD34^−^ leukemic cell lines or primary AML cells (Kobune et al., 2009; Long et al., 2016). Interestingly, the targeting of GLI1 with GANT61 resulted in myeloid differentiation of the CD34^−^ AML cell fraction (Long et al., 2016). Further evidence for the contribution of HH signaling in drug resistance was obtained by Zahreddine and others who analyzed leukemic blasts from relapsed patients treated with ribavirin, an inhibitor of the eukaryotic translation initiation factor eIF4E. In this study, UDP glucuronosyltransferase (UGT1A), an enzyme capable of inactivating ribavirin and cytarabine by glucuronidation and increasing chemoresistance, was significantly upregulated in a GLI1-dependent manner (Zahreddine et al., 2014).

Activation of the HH pathway, characterized by increased expression of SMO and GLI1, was identified in myeloid cell lines with acquired radiation and drug resistance. Upon inhibition of HH signaling with the SMO antagonist sonidegib (LDE225), these cells were resensitized to irradiation by downregulation of the PI3K/AKT/NFκB and DNA repair pathways (Li et al., 2016). This is in line with a recent study by Zhou et al. showing that overexpression of GLI1 promotes chemotherapy resistance and leukemic cell proliferation via upregulation of cell cycle regulators, such as cyclin D and cyclin-dependent kinases (CDK) and PI3K/AKT signaling. Combined inhibition of GLI1 and CDK4/6 synergistically promoted cytarabine sensitivity in cell lines and AML patient samples. RNA sequencing data from relapsed AML patient-derived bone marrow samples further confirmed that GLI1, PTCH1, SMO, and components of the PI3K/AKT signaling cascade were upregulated in relapsed AML patients compared to AML patients achieving complete remission. Furthermore, patients with high expression of GLI1, PIK3R1, and AKT3 had reduced overall survival (Zhou et al., 2021). In contrast to these studies highlighting that increased GLI1 expression contributes to AML pathophysiology, GLI3 expression seems to be actively downregulated in AML patients by epigenetic silencing to not only reduce GLI3^R^ HH repressor function (Chaudhry et al., 2017) but also upregulate the cytarabine chemoresistance-inducing genes SAMHD1, CDA, and MRP8 (Freisleben et al., 2020). HMA decitabine-induced GLI3^R^ re-expression resulted in decreased GLI1 expression levels in AML cell lines and increased their sensitivity to the SMO inhibitor glasdegib, thereby reducing AML cell viability and proliferation (Chaudhry et al., 2017). Further studies supporting the idea of dual inhibition of HH and epigenetic regulators suggest combination therapy of the histone deacetylase inhibitor vorinostat and the SMO inhibitor SANT-1 or the BRD4 inhibitor ZEN-3365 and the GLI1-inhibitor GANT61, both resulting in increased apoptosis and reduced proliferation of AML cells (Hay et al., 2017; Wellbrock et al., 2021) (Figure 2).

### Preclinical *in vivo* studies targeting Hedgehog signaling in AML

Using AML xenograft models, it was shown that *in vivo* application of glasdegib was unable to eradicate the bulk tumor cells but specifically targeted re-transplantable and self-renewing LSCs. Dissecting the underlying mechanism *in vitro*, Fukushima et al. showed that glasdegib reduces the LSC-containing CD34^+^CD38^−^ population and increases the proliferative cell fraction in patient-derived AML samples. Glasdegib treatment also resensitized MOLM-14 AML cells to cytarabine and the FLT3-kinase inhibitor sunitinib (Fukushima et al., 2016). Similarly, the GLI1 inhibitor GANT61 in combination with the FLT3-kinase inhibitor sunitinib and PI3K-inhibitor PF-04691502 was shown to downregulate GLI1 expression in and reduce the proliferation and survival of FLT3-mutated AML cells, thereby prolonging the survival of AML xenografts (Latuske et al., 2017). Conclusively, in a FLT3-ITD-driven AML mouse model concomitant, constitutive activation of HH signaling promoted the expansion of myeloid HPCs via activation of STAT5 signaling leading to accelerated AML development. Combined SMO and FLT3 kinase inhibition using saridegib and sorafenib curbed leukemic cell growth and prolonged AML mouse survival (Lim et al., 2015) (Figure 2).

Apart from the well-established GLI and SMO inhibitors, the naturally occurring small molecule triptonide was recently shown to downregulate GLI2 and FLT3 protein expression and to induce apoptosis and inhibit proliferation of FLT3-ITD^+^ AML cells in a dose dependent manner. Treatment of MOLM-13 transplanted AML xenografts with increasing dosages of triptonide significantly reduced the *in vivo* tumor burden (Xu et al., 2021).

In summary, these studies demonstrate that increased HH pathway activation is associated with poor prognosis and increased resistance to conventional treatment approaches in AML. Therefore, rational combinations of HH inhibitors with established therapeutic agents targeting the leukemic cell population represent a promising strategy to improve CR rates in chemonaïve and relapsed AML patients.

### Clinical trials targeting Hedgehog signaling in AML

Therapeutic targeting of HH signaling has been explored for various cancer entities over the last decades with most studies focusing on the inhibition of SMO. The first discovered SMO inhibitor was the naturally occurring alkaloid cyclopamine that has been extensively studied but failed clinical entrance due to poor solubility, bioavailability, and off-target effects. Intensive efforts to improve pharmacokinetics facilitated the development of clinically suited SMO antagonists such as vismodegib, sonidegib and glasdegib (Jamieson et al., 2020).

#### Vismodegib

Vismodegib (Erivedge, GDC-0449) was the first HH pathway inhibitor approved by the FDA for the treatment of locally advanced or metastatic BCC in 2012 (Axelson et al., 2013). A single-arm, open-label phase Ib study assessing the safety and efficacy of vismodegib in patients with relapsed AML was terminated due to lack of efficacy (NCT01880437) (Table 1). Although the drug was well-tolerated, with the most common adverse events (AE) being fever, nausea, taste distortion and nosebleed, all patients discontinued treatment because of disease progression (Bixby et al., 2019). Study failure may be attributed to the use of vismodegib as a single agent as preclinical studies suggested a beneficial effect of vismodegib administration during chemotherapy. Despite promising *in vitro* data (Zahreddine et al., 2014), no data from clinical studies combining vismodegib with chemotherapy in AML patients is available to date.

**TABLE 1:** SMO inhibitors in clinical trials for the treatment of AML.

SMO inhibitor	SMO inhibitor dosage	Combination	(Preliminary) Results	Clinical trial	Phase	Status	References
**Vismodegib**	150 mg/day	Ribavirin, decitabine	—	NCT02073838	II	Unknown	—
	150 mg/day	(Cytarabine)	Vismodegib well-tolerated; lower-than-expected efficacy for vismodegib monotherapy; no combination therapy performed	NCT01880437	Ib/II	Terminated	Bixby et al. (2019)
	Not defined	Not defined	—	NCT03878524	Ib	Recruiting	—
**Sonidegib**	2 × 400 mg/day or 800 mg/day	—	100% of patients experienced ≥1 AE, 71% of patients experienced serious AEs; low CR rate (1.45% of patients), disease progression in 62% of patients	NCT01826214	II	Completed	—
	400 mg/day	Azacitidine	AEs within the expected range; remission rates comparable to azacitidine monotherapy, promising progression-free and overall survival	NCT02129101	I/Ib	Completed	Tibes et al. (2017)
**Glasdegib**	5, 10, 20, 40, 80, 120, 180, 270, 400 or 600 mg/day	-	Established MTD: 400 mg/day; AEs within the expected range; two patients experienced DLT (80 and 600 mg/day); clinical activity in 49% of patients	NCT00953758	I	Completed	Martinelli et al. (2015a)
	25, 50 or 100 mg/day	LDAC, azacitidine, cytarabine/daunorubicin	Glasdegib + LDAC, well-tolerated; glasdegib + azacitidine, well-tolerated; glasdegib + cytarabine/daunorubicin, manageable despite increased severe AEs; low remission rates in glasdegib only cohort, increased in combination cohorts	NCT02038777	I	Active	Minami et al. (2017)
	Ib: 100 or 200 mg/day; II: 100 mg/day	LDAC, decitabine, cytarabine/daunorubicin	Phase Ib: glasdegib in combination with LDAC or decitabine, well-tolerated; glasdegib + cytarabine/daunorubicin, increased severe AEs, manageable; 31% remission rate across all groups; Phase II: Glasdegib + cytarabine/daunorubicin, high CR rate; glasdegib + LDAC, drastically improved CR rate and OS in combination compared to LDAC only (17 vs 2.3% and 8.8 vs. 4.9 months)	NCT01546038	Ib/II	Completed	Cortes et al. (2016); Cortes et al. (2018); Savona et al. (2018); Cortes et al. (2019b)
	100 mg/day	Azacitidine	Well-tolerated; 20% of AML patients achieved CR	NCT02367456	Ib	Completed	Sekeres et al. (2019)
	—	Cytarabine/daunorubicin, azacitidine	No improvement of OS through addition of glasdegib	NCT03416179	III	Completed	Cortes et al. (2019a)
	50, 75 or 100 mg/day	Azacitidine	—	NCT04842604	III	Active	—
	Not defined	Bosutinib, decitabine, enasidenib, ivosidenib, venetoclax, gilteritinib	—	NCT04655391	Pilot/Ib	Withdrawn	—
	100 mg/daily	CPX-351 (cytarabine/daunorubicin)	—	NCT04231851	II	Recruiting	—
	100 mg/day	Gemtuzumab ozogamicin	—	NCT04168502	III	Recruiting	—
	100 mg/day	Decitabine	—	NCT04051996	II	Terminated	—
	100 mg/day	Gemtuzumab ozogamicin	—	NCT04093505	III	Recruiting	—
	Not defined	Gemtuzumab ozogamicin	—	NCT03390296	Ib/II	Active	—
	100 mg/day	Decitabine, azacitidine, venetoclax	—	NCT03226418	II	Recruiting	—

AE, adverse event; CR, complete remission; DLT, dose-limiting toxicity; LDAC, low-dose cytarabine; MTD, maximum-tolerated dose; OS, overall survival.

#### Sonidegib

Sonidegib (Erismodegib, LDE225, Odomzo) is the second SMO inhibitor approved for treatment of advanced BCC and is currently being investigated for treatment of other cancers (Casey et al., 2017). Although *in vitro* assays demonstrated a synergistic effect between sonidegib and azacytidine in AML, combination treatment resulted in promising progression-free and overall patient survival rates but surprisingly did not enhance complete remission rates in AML patients in a phase I/Ib study (NCT02129101) (Tibes et al., 2015; Tibes et al., 2017). In a phase II study evaluating the efficacy, safety, and tolerability of sonidegib in relapsed AML (NCT01826214), all patients experienced at least 1 AE and over 70% of the patients experienced serious AEs such as febrile neutropenia, general physical health deterioration, pyrexia, asthenia, anemia, pneumonia, sepsis, increased creatine phosphokinase, and epistaxis. While around 18% of patients discontinued treatment due to these AEs, 62% of patients had a progressing AML under sonidegib treatment, which also resulted in treatment termination.

#### Glasdegib

After clinical trials for the use of previously approved SMO inhibitors in AML therapy failed, glasdegib (PF-04449913, Daurismo) in combination with low-dose cytarabine (LDAC) was the first SMO inhibitor to receive FDA approval for the treatment of AML at the end of 2018.

As determined during safety and pharmacokinetic profiling of orally applied glasdegib, the maximum tolerated dose was 400 mg per day with a mean plasma half-life of 24 h. In light of the commonly observed AEs, such as taste distortion, decreased appetite and hair loss, the recommended dose was lowered to below 200 mg per day (NCT00953758) (Martinelli et al., 2015b). Combination of glasdegib with standard of care treatments was assessed in a phase Ib study (NCT01546038) in patients with AML or high-risk MDS. Newly diagnosed patients ineligible for intensive induction therapy received glasdegib with LDAC (group A) or decitabine (group B), whereas fit patients received glasdegib with cytarabine and daunorubicin (group C). The CR/CR with incomplete blood count recovery (CRi) rate was 31% across all patients, 9% in group A, 29% in group B and 55% in group C. Median overall survival (OS) was 4.4 months in group A, 11.5 months in group B and 34.7 months in group C. All treatment arms were generally well-tolerated with low grade AEs. Whereas most AEs were consistent with AML-related complications during standard-of-care therapy, glasdegib treatment-related muscle spasms, dysgeusia, and alopecia were also observed. Of note and in line with the clinical trial NCT02038777, severe AEs were frequently observed but manageable in patients receiving glasdegib in combination with cytarabine and daunorubicin. As the maximum administered dose (MTD) was not reached for glasdegib within this study, the recommended phase II dose for glasdegib of 100 mg per day was based on the observed tolerability for the combination therapy (glasdegib + LDAC or intensive chemotherapy), successful inhibition of HH signaling, and glasdegib pharmacokinetics (Savona et al., 2018). A subsequent phase II study (NCT01546038) evaluating the efficacy of 100 mg daily glasdegib administered in combination with standard cytarabine and daunorubicin chemotherapy in patients with untreated AML or high-risk MDS revealed that 46.4% of all patients achieved CR and increased the median OS to 14.9 months. While the most common treatment-related AEs were tolerable diarrhea and nausea, around 20% of patients had to discontinue treatment due to treatment-related pneumonia or sepsis. Importantly, there were no significant associations between recurrent AML mutations and clinical response (Cortes et al., 2018). Unfortunately, preliminary data from a phase III trial investigating the outcome of patients receiving glasdegib in combination with cytarabine and daunorubicin suggest no improvement of patient overall survival (17.3 months OS for glasdegib + cytarabine/daunorubicin and 20.4 months OS for cytarabine/daunorubicin; NCT03416179). In a simultaneously conducted second phase II study (BRIGHT AML 1003, NCT01546038) LDAC with or without glasdegib was evaluated in patients with untreated AML or high-risk MDS ineligible for intensive chemotherapy. 20 mg LDAC was administered on 10 days per 28-days cycle with or without 100 mg glasdegib daily for 28 days. Impressively, 15 patients (17%) achieved CR under LDAC + glasdegib therapy compared to only 1 patient (2.3%) in the LDAC-only group. Median OS was 8.8 and 4.9 months for LDAC + glasdegib and LDAC treatment, respectively. The most common non-hematologic sever AEs were pneumonia, fatigue, dyspnea, hyponatremia, sepsis and syncope in the LDAC + glasdegib arm and pneumonia in the LDAC-only arm. Despite the high frequency of sever AEs (80%), the safety profile was considered manageable and a favorable benefit-risk profile was demonstrated with this study, thereby leading to the FDA approval of glasdegib in combination with LDAC for the treatment of newly diagnosed AML patients unsuitable for intensive induction chemotherapy (Cortes et al., 2016; Cortes et al., 2019b). Post hoc analysis of this study further revealed a clinical benefit even for LDAC + glasdegib treated patients who did not achieve CR and confirmed the prolonged median OS for patients who achieved CR (26.1 months LDAC + glasdegib; 12.9 months LDAC-only) (Cortes et al., 2020). Noteworthy, the superior clinical efficacy of LDAC combined with glasdegib compared to LDAC-only was observed for all cytogenetic risk groups. Furthermore, a subgroup analysis revealed a more pronounced survival benefit of LDAC + glasdegib in patients with secondary AML and relapsed AML patients not receiving HMA therapy (Heuser et al., 2021). Overall, the BRIGHT AML 1003 trial applying LDAC in combination with glasdegib demonstrated impressive clinical efficacy in difficult to treat patients. Further studies evaluating the combination of glasdegib with various drugs are ongoing or planned (Table 1). Hence the acquisition of resistance mechanisms upon HMA monotherapy and the success of combination treatments with an HMA backbone, the results of trials combining SMO inhibitors with either azacitidine or decitabine are eagerly awaited. Preliminary data obtained from studies (NCT02367456 and NCT03416179) evaluating clinical efficacy of glasdegib in combination with azacitidine in untreated AML patients suggested improved CR rates. However, first analysis of patient overall survival data revealed no beneficial effect of adding glasdegib (10.3 months OS for glasdegib + azacitidine and 10.6 months OS for azacitidine) (Cortes et al., 2019a; Sekeres et al., 2019). A more detailed analysis of the partially available data is required for further conclusions.

The combination of glasdegib with already established inhibitors targeting IDH1/2, BCL-2 or FLT3 in AML are obviously of high interest and have been incorporated into a clinical trial draft (NCT04655391). Unfortunately, this study was stopped prior to patient enrollment due to limited drug availability. An additional AML study investigating the combinational effect of glasdegib with, e.g., venetoclax on CR rate and mortality is currently recruiting patients (NCT03226418).

## Conclusion and outlook

Adapting AML treatment to a patient’s individual needs requires a growing arsenal of small molecule inhibitors, HMAs, and chemotherapeutic agents to simultaneously target multiple oncogenic pathways and tumor-promoting processes. As reviewed here, successful preclinical and clinical AML studies inhibiting HH signaling have recently led to the FDA approval of the SMO inhibitor glasdegib in combination with LDAC for the treatment of newly diagnosed and unfit AML patients. Ongoing clinical trials are further evaluating the benefit of SMO inhibitor combination therapies with HMAs, IDH inhibitors, tyrosine kinase inhibitors and/or pyrimidine analogues. These studies will reveal if and how AML patient subgroups benefit from HH inhibiting treatment approaches and position glasdegib and other HH inhibitors in the landscape of AML therapy. Furthermore, combination therapies targeting canonical and non-canonical HH signaling simultaneously are envisioned and might offer new therapeutic approaches preventing the development of drug resistance. Although the clinical efficacy of glasdegib is undeniable, the exact tumor targeting mechanism of HH signal inhibition in AML is still not clear. Whether the anti-leukemic effects are exclusively attributable to abrogating AML (stem) cell intrinsic HH signaling or to blocking HH signaling within the tumor supporting bone marrow microenvironment remains to be clarified and calls for a more detailed analysis of HH signaling components and regulators in AML patient-derived blasts, LSCs and bone marrow stromal and endothelial cells. This will also be crucial for the identification of biomarkers predictive for HH inhibitor response of AML patients.
